# Machine learning-based radiomics for predicting BRAF-V600E mutations in ameloblastoma

**DOI:** 10.3389/fimmu.2023.1180908

**Published:** 2023-08-14

**Authors:** Wen Li, Yang Li, Xiaoling Liu, Li Wang, Wenqian Chen, Xueshen Qian, Xianglong Zheng, Jiang Chen, Yiming Liu, Lisong Lin

**Affiliations:** ^1^ Department of Oral and Maxillofacial Surgery, The First Affiliated Hospital of Fujian Medical University, Fuzhou, China; ^2^ School and Hospital of Stomatology, Fujian Medical University, Fuzhou, China; ^3^ Department of Oral and Maxillofacial Surgery, The First Affiliated Hospital of Zhengzhou University, Zhengzhou, China

**Keywords:** ameloblastoma, machine learning, radiomics, LASSO, BRAF-V600E

## Abstract

**Background:**

Ameloblastoma is a locally invasive and aggressive epithelial odontogenic neoplasm. The BRAF-V600E gene mutation is a prevalent genetic alteration found in this tumor and is considered to have a crucial role in its pathogenesis. The objective of this study is to develop and validate a radiomics-based machine learning method for the identification of BRAF-V600E gene mutations in ameloblastoma patients.

**Methods:**

In this retrospective study, data from 103 patients diagnosed with ameloblastoma who underwent BRAF-V600E mutation testing were collected. Of these patients, 72 were included in the training cohort, while 31 were included in the validation cohort. To address class imbalance, synthetic minority over-sampling technique (SMOTE) is applied in our study. Radiomics features were extracted from preprocessed CT images, and the most relevant features, including both radiomics and clinical data, were selected for analysis. Machine learning methods were utilized to construct models. The performance of these models in distinguishing between patients with and without BRAF-V600E gene mutations was evaluated using the receiver operating characteristic (ROC) curve.

**Results:**

When the analysis was based on radiomics signature, Random Forest performed better than the others, with the area under the ROC curve (AUC) of 0.87 (95%CI, 0.68-1.00). The performance of XGBoost model is slightly lower than that of Random Forest, and its AUC is 0.83 (95% CI, 0.60-1.00). The nomogram evident that among younger women, the affected region primarily lies within the mandible, and patients with larger tumor diameters exhibit a heightened risk. Additionally, patients with higher radiomics signature scores are more susceptible to the BRAF-V600E gene mutations.

**Conclusions:**

Our study presents a comprehensive radiomics-based machine learning model using five different methods to accurately detect BRAF-V600E gene mutations in patients diagnosed with ameloblastoma. The Random Forest model’s high predictive performance, with AUC of 0.87, demonstrates its potential for facilitating a convenient and cost-effective way of identifying patients with the mutation without the need for invasive tumor sampling for molecular testing. This non-invasive approach has the potential to guide preoperative or postoperative drug treatment for affected individuals, thereby improving outcomes.

## Introduction

Ameloblastoma is a common benign tumor of dental origin, arising from the epithelial component of the developing dental embryo, and often affecting the mandible or maxilla (1) The BRAF-V600E gene mutation is frequently reported in approximately 70% of ameloblastoma (2). Ameloblastoma typically grows slowly but may show features of local invasion into surrounding tissues or cause bone resorption (3). Surgical excision is the most commonly used treatment approach due to the tumor’s complex growth pattern, but it can lead to facial deformities (4) and disease recurrence (5). On the other hand, conservative approaches such as fenestration decompression combined with secondary curettage and local curettage, lead to a high rate of tumor recurrence, and radical excisional surgery remains the preferred treatment option (6, 7). The transformation of ameloblastoma to ameloblastic carcinoma, although rare in clinical practice, is still a priority for clinicians when diagnosing the disease (8). Moreover, the recurrence of ameloblastoma can extend over many years, and a disease-free period of 5 years does not necessarily imply complete recovery (9). Therefore, regular follow-up is required. In summary, there is a pressing need for targeted treatment modalities for this disease to avoid extensive surgery and disease recurrence. The pathogenesis of ameloblastoma at the molecular level is not yet fully understood. However, some studies have identified potential prognostic markers or therapeutic targets, indicating the need for further research (2). Interestingly, genetic molecular alterations in ameloblastoma have been shown to be associated with clinical features and patient prognosis (10, 11). The BRAF-V600E gene mutation has been identified as a crucial factor in the pathogenesis of ameloblastoma (12). The MAPK/ERK pathway has been found to be activated by the BRAF-V600E gene mutation (13), leading to increased cell proliferation and inhibition of apoptosis and promoting the development of ameloblastoma (14). The prevalence of BRAF-V600E gene mutation in aggressive and recurrent ameloblastoma suggests a potential role in the biological behavior of the tumor (15). Other molecular mechanisms, such as the activation of the Wnt/β-catenin pathway, have also been implicated in the pathogenesis of ameloblastoma, leading to increased cell proliferation and inhibition of apoptosis (16). Further investigation into these pathways may provide important insights into the development and progression of ameloblastoma and facilitate the development of targeted therapies.

Radiomics is an increasingly important discipline in the medical field, providing quantitative analysis of medical images using advanced computational techniques to extract multiple features and increase the accuracy of clinical decision making by physicians (17, 18). Radiomics analysis has been applied to predict mutations in tumor somatic cells in various cancers (19). Yang et al. (20) showed good area under the curve (AUC) and specificity in predicting KRAS/NRAS/BRAF gene mutations in colorectal cancer patients based on radiomics features of computed tomography (CT). Radiomics has also been used to predict response to chemotherapy and radiotherapy in non-small cell lung cancer patients, assisting clinicians in making treatment decisions (21). While some studies have explored the relationship between radiomics features and BRAF gene mutation status, the role of CT-based machine learning (ML) for radiomics in identifying BRAF-V600E gene mutations in ameloblastoma remains to be investigated.

Therefore, the objective of this study is to develop a radiomics-based model that predicts the BRAF-V600E gene mutation status in patients with ameloblastoma. This study will employ five ML algorithms based on CT and combine radiomics and clinical features of patients to evaluate the model’s effectiveness in predicting BRAF-V600E gene mutations in ameloblastoma. The outcomes of this study may prove valuable in distinguishing patients with ameloblastoma who have developed BRAF-V600E gene mutations, and help clinicians make treatment decisions without resorting to invasive testing.

## Methods

### Patients

**Figure 1:**
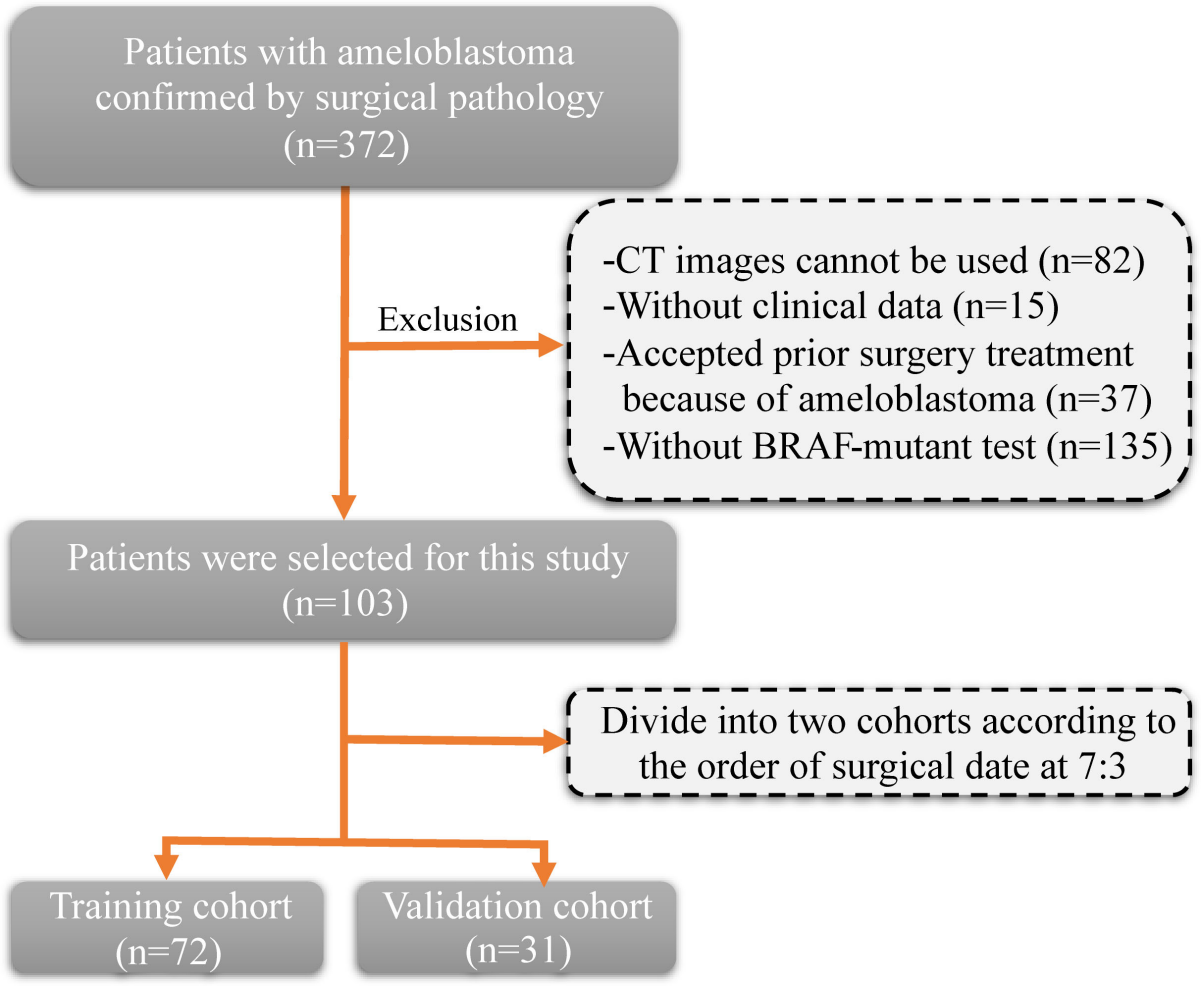
Flow diagram of the study population.

The inclusion criteria and procedures for participant recruitment in this retrospective study were in accordance with the guidelines stipulated in the 1964 Helsinki Declaration. Approval for this study was obtained from the Ethics Committee of the First Affiliated Hospital of Zhengzhou University (approval number: 2023-KY-0140). One hundred and three patients diagnosed with ameloblastoma at the First Affiliated Hospital of Zhengzhou University between January 2012 and December 2022 were included in this study. The clinical information of patients includes age, gender, tumor site, and tumor diameter. The inclusion criteria were as follows: (1) a pathological diagnosis of ameloblastoma; (2) clear CT images prior to surgery; and (3) detection of the BRAF-V600E gene mutation after surgery. The exclusion criteria (**Figure 1**) were as follows: (1) CT images cannot be used (n=82); (2) without clinical data (n=15); (3) without BRAF-mutant test (n=135); and (4) accepted prior surgery treatment because of ameloblastoma (n=37). Patients were allocated into two distinct groups, namely a training cohort and a validation cohort, based on the chronological sequence of their surgical procedures. The training cohort comprised 72 patients who underwent surgery within the timeframe spanning January 2012 to January 2020. On the other hand, the validation cohort incorporated 31 patients whose surgical procedures took place between February 2020 and December 2022.

### Image acquisition and processing

The CT machine utilized in this study was the Aquilion 16 CT (Toshiba, Japan), located at the First Affiliated Hospital of Zhengzhou University. The following scan parameters were employed: voltage 120kV, current 200 mA, and a slice thickness of 5mm.

### Regions of interest (ROI) segmentation and mask dilation

**Figure 2:**
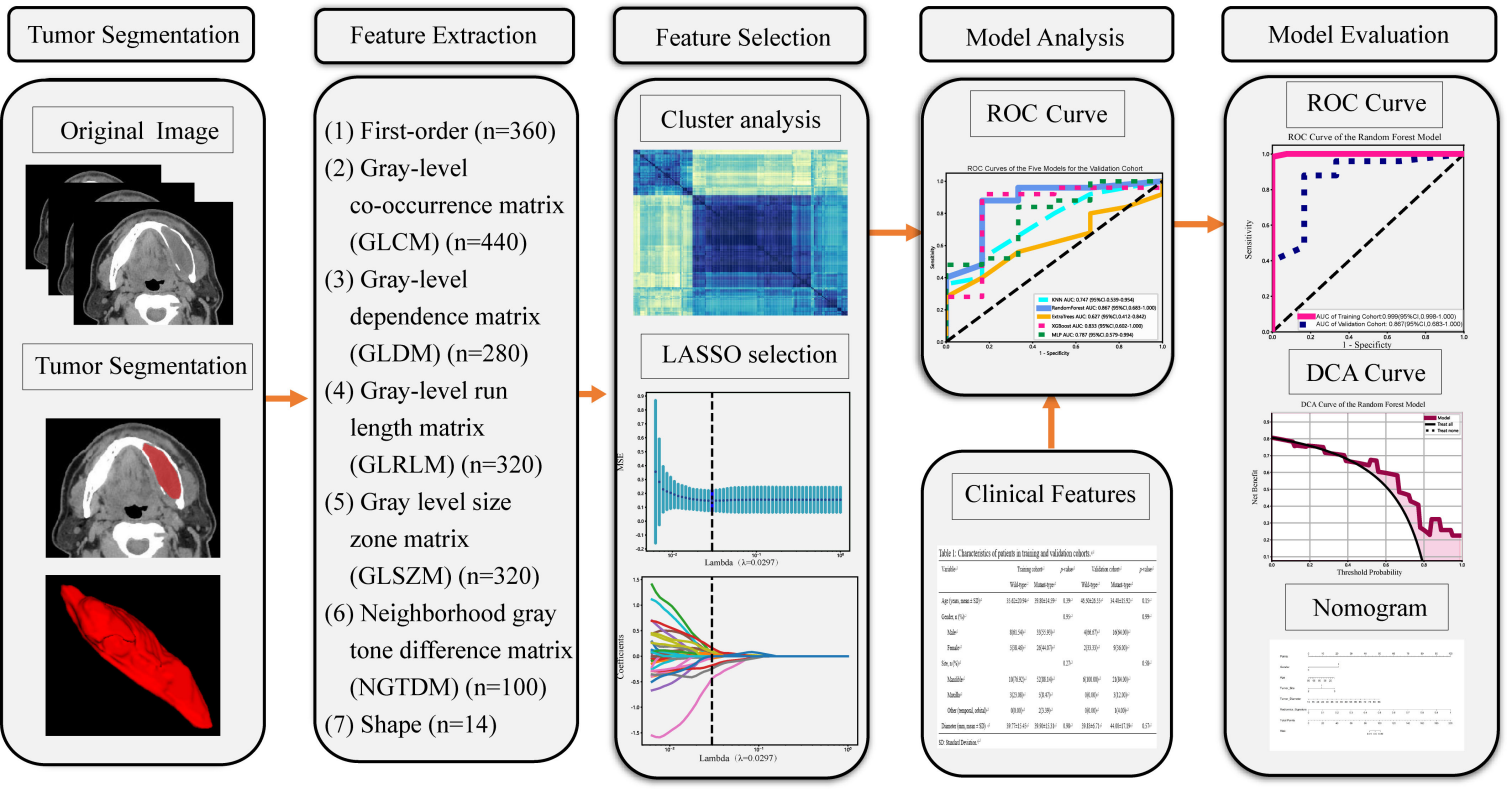
Workflow of the study. LASSO, the least absolute shrinkage and selection operator; n, Number of features; ROC, receiver operating characteristic; DCA, decision curve analysis.

Our radiomics analysis encompassed various stages: lesion segmentation, feature extraction, feature selection, and feature analysis and evaluation (**Figure 2**). Prior to initiating the lesion segmentation, we implemented a resampling process to standardize the images in accordance with the Image Biomarker Standardization Initiative (IBSI) guidelines (22), which involved resampling voxel sizes of 1 mm × 1 mm × 1 mm. The patient CT images collected for this study were obtained at a resolution of 512 × 512.

ROIs were manually segmented on a slice-by-slice basis along the lesions utilizing ITK-SNAP software (version 3.8.0, www.ITK-SNAP.org). An oral surgeon with over three years of experience undertook this segmentation, with the individual blinded to the clinical information of the patients. Another senior dentist with five years of experience verified all manual delineations. The delineated ROIs were saved in Neuroimaging Informatics Technology Initiative (NII) format for further analysis. Subsequently, quantitative radiomic features were extracted from CT images, using Pyradiomics software (version 2.2.0, http://pyradiomics.readthedocs.io) (23). The intraclass correlation coefficients (ICCs) were calculated to gauge the consistency between the features extracted by the two radiologists. Any features presenting intra-observer or inter-observer ICCs less than 0.75 were excluded, attributable to their comparatively low robustness (24).

### Analysis of BRAF-V600E gene mutations

Based on the specimen, BRAF-V600E gene mutations were examined using real-time fluorescent polymerase chain reaction (PCR) and DNA sequencing (ABI Step One/ABI sequence Analyzer) technologies (25). The nucleic acid’s original concentration was 184 ng/μl. In the present study, wild-type BRAF-V600E referred to the absence of mutations in those loci.

### Radiomics feature extraction

The hand-crafted features can be categorized into three groups: (1) geometric features, (2) intensity features, and (3) texture features. Extracted features comprised 360 first-order features, 440 gray-level co-occurrence matrix (GLCM) features, 280 gray-level dependence matrix (GLDM) features, 320 gray-level run length matrix (GLRLM) features, 320 gray-level size zone matrix (GLSZM) features, 100 neighboring gray tone difference matrix (NGTDM) features and 14 shape features (**Supplementary Datasheet 1**). In total 1834 radiomics features were extracted from ROIs.

### Feature selection

Prior to in-depth analysis, all extracted radiomics features underwent standardization into a normal distribution using z-scores, thereby nullifying potential discrepancies in data value scales. Given the contrast between the relatively low dimensional sample size and the high dimensional radiomics features, feature selection was indispensable to prevent overfitting (26). We conducted a Student’s t-test for features adhering to a normal distribution, only considering features with a *p*-value less than 0.05 for subsequent analysis.

Spearman’s rank correlation coefficient was used to assess the correlation between features with high repeatability (27). To avoid redundancy, we retained only one feature from any pair with a correlation coefficient greater than 0.9 (28). To maximize the informative value of the feature set, we employed a greedy recursive deletion strategy for feature filtering. This involved removing the feature with the greatest redundancy in the current set until 51 features remained.

We employed the Least Absolute Shrinkage and Selection Operator (LASSO) regression model to construct a signature based on the discovery dataset. LASSO regression shrinks all regression coefficients towards zero and sets many coefficients of uncorrelated features to exactly zero. The optimal regularization weight λ was determined using a minimum criterion and 10-fold cross-validation. Retained features with non-zero coefficients were used to fit the regression model and combined to form radiomics features. A radiomics score was then calculated for each patient by weighting the linear combination of the retained features by their model coefficients. We used the Python *scikit-learn* package (29) for LASSO regression modeling.

### Radiomics signature

Radiomics data were balanced using synthetic minority over-sampling technique (SMOTE) algorithm synthesis (30). In this work, we processed hyperparameter optimization using grid search to optimize the parameters of models and apply the best parameters to predict BRAF-V600E gene mutations in ameloblastoma. After LASSO feature screening, the final features were input into various ML models, including K-Nearest Neighbor (KNN), Random Forest, ExtraTrees, eXtreme Gradient Boosting (XGBoost) and Multilayer Perceptron (MLP), for constructing the risk model. To obtain the final radiomics signature, a 5-fold cross-validation approach was adopted. Radiomics-clinical nomogram was developed by combining the radiomics signature and clinical features using the logistic regression algorithm.

### Statistical analysis

In an endeavor to ascertain the equivalence of patient attributes across cohorts, we applied differing statistical approaches for data analysis. Student’s t-test were utilized for the analysis of normally distributed data, whilst non-normally distributed data were scrutinized using the Mann-Whitney U test. For categorical variables, chi-square tests proved the method of choice for evaluation. Furthermore, we conducted an assessment of the predictive power of three distinctive models using ROC curves. The calculation of the AUC was undertaken, followed by the computation of the balanced sensitivity and specificity of the cut-off point, yielding the maximum value of the Youden index. We calculated the 95% confidence interval (CI) of the AUC utilizing the bootstrap method with 1000 intervals for increased precision. This comprehensive and rigorous analysis approach serves to illuminate the strengths and potential limitations of our study, providing a more robust understanding of the data and underlying patterns therein. The AUC ranged from 0.5 to 1.0. The discriminative test was deemed perfect when the AUC equaled 1.0. An AUC between 0.8 and 1.0 was indicative of a good discriminant test, whereas an AUC ranging from 0.6 to 0.8 represented a moderate test. If the AUC fell within the 0.5 to 0.6 range, the discriminant test was considered poor (31, 32). We performed statistical analyses using SPSS software (version 21.0). A two-sided *p*-value of less than or equal to 0.05 was stipulated as the threshold for statistical significance.

## Results

### Clinical characteristics

The baseline clinical characteristics of the enrolled patients are presented in **Table 1**. No significant differences were observed in terms of age, gender, tumor site, and tumor diameter between gene mutation and non-mutation groups (*p*-value > 0.05). There was no statistically significant difference (*p*-value > 0.05) between clinical characteristics and predicted BRAF-V600E mutation on univariate analysis (**Supplementary Table 1**).

**Table 1:** Characteristics of patients in training and validation cohorts.

Variable	Training cohort		*p*-value	Validation cohort		*p*-value
	Wild-type	Mutant-type		Wild-type	Mutant-type	
Age (years, mean ± SD)	35.62±20.94	39.80±14.59	0.39	46.50±26.55	34.40±15.92	0.15
Gender, n (%)			0.95			0.99
Male	8 (61.54)	33 (55.93)		4 (66.67)	16 (64.00)	
Female	5 (38.46)	26 (44.07)		2 (33.33)	9 (36.00)	
Site, n (%)			0.27			0.58
Mandible	10 (76.92)	52 (88.14)		6 (100.00)	21 (84.00)	
Maxilla	3 (23.08)	5 (8.47)		0 (0.00)	3 (12.00)	
Other (temporal, orbital)	0 (0.00)	2 (3.39)		0 (0.00)	1 (4.00)	
Diameter (mm, mean ± SD)	39.77±15.45	39.90±15.31	0.98	39.83±6.71	44.00±17.19	0.57

SD, Standard Deviation.

### LASSO feature selection

**Figure 3:**
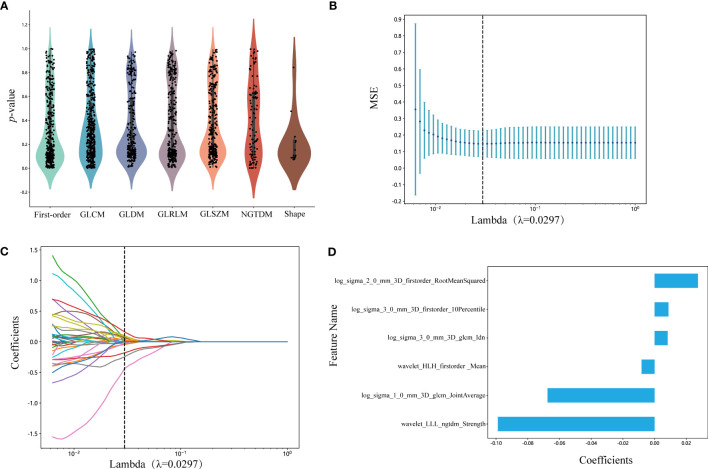
**(A)** Statistics of radiomic features. Points represent features. GLCM, gray-level co-occurrence matrix; GLDM, gray-level dependence matrix; GLRLM, gray-level run length matrix; GLSZM, gray-level size zone matrix; NGTDM, neighboring gray tone difference matrix. **(B)** Mean square error of cross-validation of LASSO model. The optimal λ value is 0.0297. MSE: mean square error. **(C)** LASSO coefficient solution path of features. The optimal λ value is 0.0297. **(D)** The histogram of the feature score. The y-axis indicates the selected six radiomics features, and the x-axis represents the coefficients of LASSO model. LASSO, the least absolute shrinkage and selection operator; LLL, low-low-low-pass filtered image; HLH, high-low-high-pass filtered image.

We selected 1834 features for the extraction of ROIs, and the categories of features and the corresponding *p*-values are shown in **Figure 3A** and in **Supplementary Datasheet 1**. We kept 155 features that the *p*-value was less or equal to 0.05. The correlation coefficients for each feature were visualized and can be seen in **Supplementary Datasheet 2**. Subsequently, six nonzero coefficient features were selected to create radiomics-scores with a LASSO logistic regression model (λ = 0.0297) (**Figures 3B, C**). The histograms of the feature scores are shown in **Figure 3D**.

### Diagnostic performance among radiomics model and nomogram

**Figure 4:**
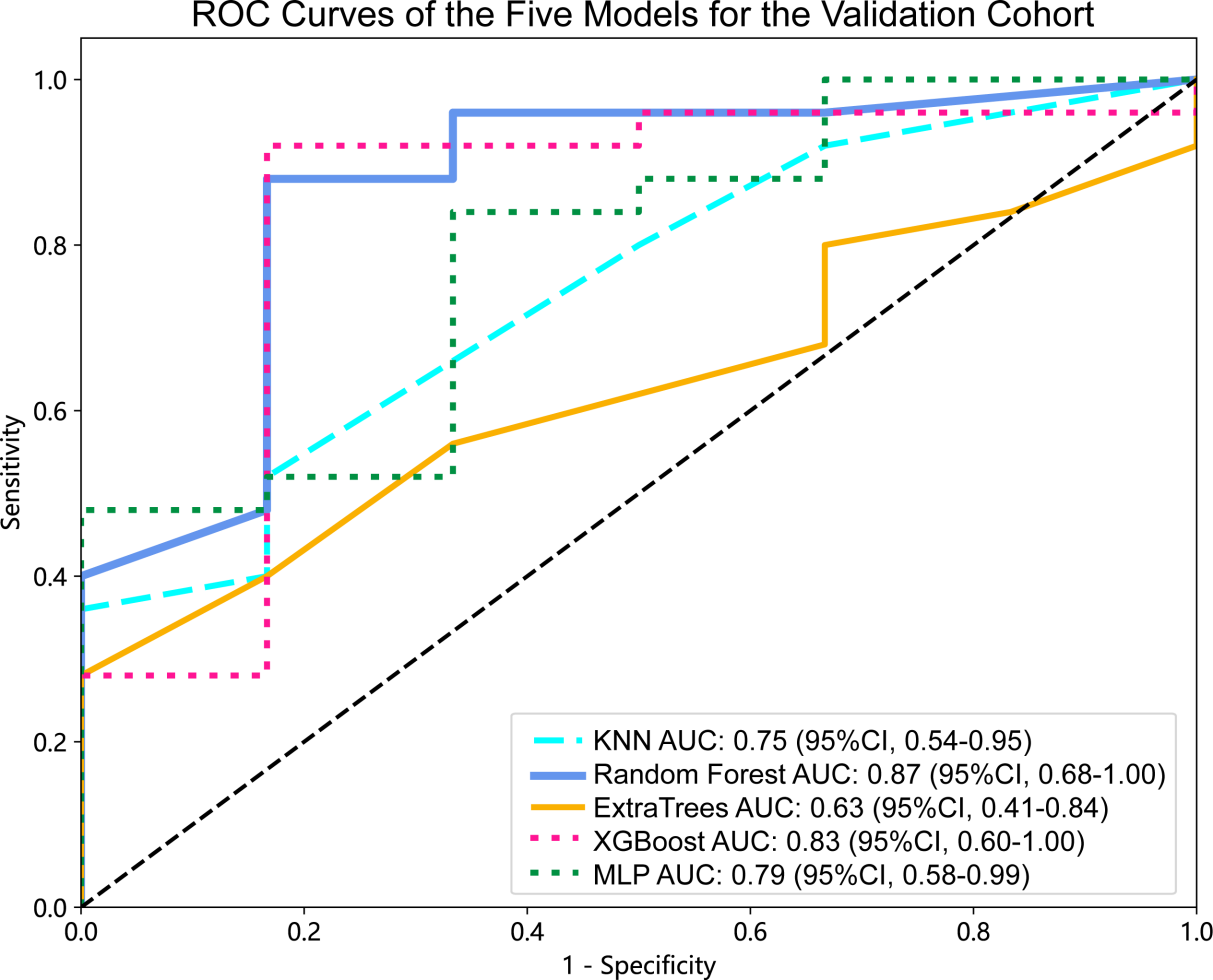
ROC curves of the five models for the validation cohort. AUC, area under ROC curve; ROC, receiver operating characteristic; CI, confidence interval; KNN, K-Nearest Neighbor; XGBoost, eXtreme Gradient Boosting; MLP, Multilayer Perceptron.

**Figure 5:**
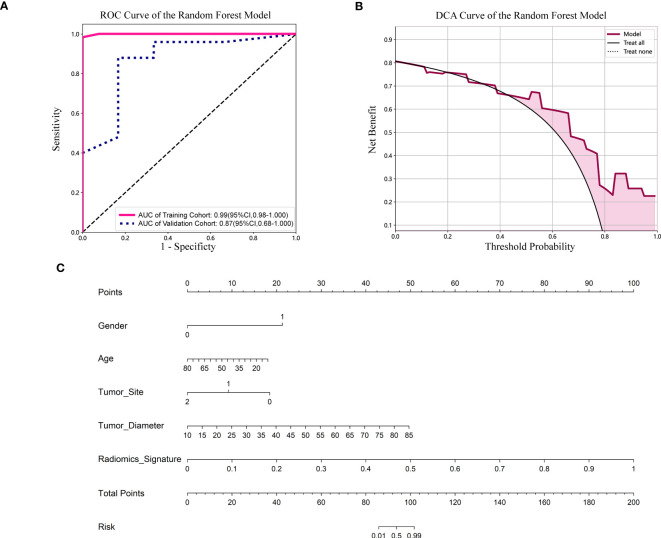
**(A)** ROC curve of the Random Forest model. CI, confidence interval; ROC, receiver operating characteristic; AUC, area under ROC curve. **(B)** DCA of the Random Forest model. DCA: decision curve analysis. **(C)** Nomogram based on the clinical and radiomics features prediction model to predict the risk of BRAF-V600E gene mutation. Gender, 0; male, 1; female. Tumor site, 0; mandible, 1; maxilla, 2; other (temporal, orbital).

For the validation cohort, the AUC value for each classifier across the different ML algorithms are presented in **Figure 4** (more details in **Supplementary Table 2**). When the analysis was based on radiomics signature, Random Forest performed better than the others, with AUC of 0.87 (95%CI, 0.68-1.00) (**Figure 5A**). The performance of XGBoost model is slightly lower than that of Random Forest, and its AUC is 0.83 (95% CI, 0.60-1.00). These two models have a good performance. The other three models performed moderately. In this study, we evaluated the model through decision curve analysis (DCA). The DCA for Random Forest model is presented in **Figure 5B**.

The nomogram combined the clinical features (gender, age, tumor site and diameter) and radiomics signature (**Figure 5C**). It is evident from **Figure 5C** that among younger women, the affected region primarily lies within the mandible, and patients with larger tumor diameters exhibit a heightened risk. Additionally, patients with higher radiomics signature scores are more susceptible to the BRAF-V600E gene mutation.

## Discussion

Ameloblastoma is a common tumor of dental origin, and its biological behavior is complex and not yet fully understood. This disease is prone to recurrence and has a tendency to become malignant, often classified as borderline tumors. Pulmonary metastases have also been reported in some cases of ameloblastoma (33). The conventional treatment for ameloblastoma is surgical resection, which depends on various factors such as tumor location, size, histological type, patient’s age, and general health (34). The goal is to achieve complete removal of the tumor while preserving the patient’s physical function and aesthetic appearance as much as possible. However, due to the slow growth of ameloblastoma, patients often present with large tumors at the time of consultation, which may result in facial deformities after resection and impair oral and maxillofacial function, leading to physical and mental health issues (35). Therefore, early detection, diagnosis, and treatment of ameloblastoma are crucial. It is also important to explore new adjuvant treatments in combination with surgery to reduce the recurrence rate of this disease. In the realm of ameloblastoma research, the focus of existing radiomics studies has largely been on the differential diagnosis of the disease. For instance, Liu et al. (36) utilized a Convolutional Neural Network (CNN) methodology to distinguish between ameloblastoma and odontogenic keratocyst, drawing on the patients’ panoramic radiographs for their analyses. Alternatively, Chai et al. (37) adopted a similar CNN modeling approach, but their study was distinctive in that it relied on patients’ cone beam computed tomography (CBCT) data to differentiate between the two conditions.

Ameloblastoma is a heterogeneous tumor that can be classified into different subtypes based on their histological characteristics, including conventional, unicystic, and desmoplastic types, among others. Each subtype exhibits distinct biological behaviors and treatment responses (38). Meanwhile, it has been found that ameloblastoma carrying mutations in the BRAF gene tend to occur more frequently in the mandible and in younger patients (39). In the era of precision medicine, it is crucial to identify the molecular features of different disease subtypes and develop targeted therapies accordingly. Previous studies have used radiomics features as the primary investigative tool to identify the molecular subtypes of various gene mutations present in low-grade gliomas (40). Multiple studies have demonstrated that the BRAF-V600E gene mutation contributes to the activation of the MAPK signaling pathway and plays a crucial role in the pathogenesis of ameloblastoma (41). Therefore, the development of BRAF-V600E-specific inhibitors represents a promising approach to improve the treatment of ameloblastoma in addition to surgery. Currently, BRAF-V600E inhibitors such as Zelboraf and Dabrafenib are approved for the treatment of melanoma (42), while their use in ameloblastoma is still under investigation. However, some studies have shown promising clinical outcomes with the use of Dabrafenib in ameloblastoma (43). As research into the BRAF-V600E gene mutation continues, the use of targeted therapeutic agents for this mutation in ameloblastoma is expected to become a valuable adjuvant treatment to reduce the recurrence rate of patients.

In this study, we propose a predictive model based on a combination of non-invasive CT images and patient clinical features to predict BRAF-V600E gene mutation status in patients with ameloblastoma. We included clinical information such as age, gender, tumor location, and tumor diameter of patients with ameloblastoma to establish a correlation between this clinical information and the BRAF-V600E gene mutation. ML algorithms were used to identify patterns and relationships in the data. Five ML models were trained on 72 patients, and their performance was validated with 31 patients. The Random Forest model performed good predictability with AUC of 0.87 (95%CI, 0.68-1.00) in the validation cohort, indicating that it may handle noisy data in CT images more effectively than other tree-based models. The performance of XGBoost model is slightly lower than that of Random Forest, and its AUC is 0.83 (95% CI, 0.60-1.00). These two models have a good performance.

The nomogram showed the importance ranking of individual features, with radiomics features having greater importance than patient clinical information, which is consistent with the results derived from the Random Forest model. This study provides evidence of a clear association between CT image features and BRAF-V600E genotype, and demonstrates the ability of radiomics to identify BRAF-V600E gene mutation status. The prediction of BRAF-V600E gene mutations based on Random Forest models has the potential to replace conventional invasive biopsies. To our knowledge, this is the first study to build a ML model to predict BRAF-V600E mutation status in patients with ameloblastoma. Therefore, this study makes a significant contribution to existing research in this field.

Supervised learning and unsupervised learning are the two main ML methods. While supervised learning has been the primary method in the field of data mining (44), all five ML models used in this study are supervised learning methods. Our retrospective study demonstrated that the Random Forest models were viable for predicting BRAF-V600E gene mutation status.

There are several limitations to this study that should be acknowledged. Firstly, being a retrospective study, it may have some inherent limitations such as data selection bias. Secondly, the sample size of patients included in the study was relatively small after a rigorous screening process. However, we believe that the inclusion of more than 100 patients for radiological analysis is desirable in the current study (45). In future studies, we plan to expand the sample size to assess the stability and clinical application of the Random Forest models. Simultaneously, we will persist in our patient follow-up efforts and utilize radiomics features to prognosticate their progression-free survival, particularly concerning recurrence of ameloblastoma, drawing inspiration from the research trajectory established by Le et al. (46). Additionally, we aim to employ semi-automated or automated radiological methods in future studies to enhance the robustness of the prediction models used in this study.

## Conclusion

In conclusion, this study demonstrated that a combination of radiomics signatures and clinical features can accurately predict BRAF-V600E gene mutation status in patients with ameloblastoma. While these findings require validation with a larger sample size, the use of machine learning models provides a non-invasive and cost-effective approach for predicting BRAF-V600E gene mutations. This approach could potentially aid in screening patients before resorting to invasive sampling and in developing personalized treatment plans to optimize outcomes for patients with ameloblastoma.

## Data availability statement

The original contributions presented in the study are included in the article/**Supplementary Material**. Further inquiries can be directed to the corresponding authors.

## Ethics statement

Approval for this study was obtained from the Ethics Committee of The First Affiliated Hospital of Zhengzhou University (approval number: 2023-KY-0140). Written informed consent from the participants’ legal guardian/next of kin was not required to participate in this study in accordance with the national legislation and the institutional requirements.

## Author contributions

WL and YaL contributed equally to this study. WL and YaL designed and wrote the main manuscript text. XL and LW collected information and analyzed data on the patients included in the study. WC, XQ and XZ edited and revised the manuscript text. JC, YiL and LL presented the research oversaw its implementation. All authors contributed to the article and approved the submitted version.
