# Supplementary material for: Machine learning-based radiomics for predicting BRAF-V600E mutations in ameloblastoma
Source: Front Immunol. 2023 Aug 14;14:1180908. doi: 10.3389/fimmu.2023.1180908 (PMC10461083; doi:10.3389/fimmu.2023.1180908)
Supplement: Supplementary file 3 [file DataSheet_3.docx]

Supplementary Material

**Machine Learning-Based Radiomics for Predicting BRAF-V600E Mutations in Ameloblastoma**

**Wen Li^1†^, Yang Li^2†^,** **Xiaoling Liu^3^, Li Wang^3^,** **Wenqian Chen^2^, Xueshen Qian^2^, Xianglong Zheng^1^, Jiang Chen ^2^; Yiming Liu^3*^, Lisong Lin^1*^**

1 Department of Oral and Maxillofacial Surgery, The First Affiliated Hospital of Fujian Medical University, Fuzhou, China. [zzlw8651@fjmu.edu.cn](mailto:zzlw8651@fjmu.edu.cn) (Wen Li); [18705007141@163.com](mailto:18705007141@163.com)(Xianglong Zheng); [dr_lls@hotmail.com](mailto:dr_lls@hotmail.com) (Lisong Lin)

2 School and Hospital of Stomatology, Fujian Medical University, Fuzhou, China. [liyang0207@fjmu.edu.cn](mailto:liyang0207@fjmu.edu.cn) (Yang Li); [chenwq_helen@163.com](mailto:chenwq_helen@163.com) (Wenqian Chen); [qianxueshenkq@fjmu.edu.cn](mailto:qianxueshenkq@fjmu.edu.cn) (Xueshen Qian); [jiangchen@fjmu.edu.cn](mailto:jiangchen@fjmu.edu.cn) (Jiang Chen)

3 Department of Oral and Maxillofacial Surgery, The First Affiliated Hospital of Zhengzhou University, Zhengzhou, China. [13663853119@163.com](mailto:13663853119@163.com) (Xiaoling Liu); [liwangattempt@163.com](file:///D:\042.博士期间的文章\liwangattempt@163.com) (Li Wang); [doctorliuym@163.com](mailto:doctorliuym@163.com)(Yiming Liu)

***Correspondence**

*Yiming Liu*

[doctorliuym@163.com](mailto:doctorliuym@163.com)

*Lisong Lin*

[dr_lls@hotmail.com](mailto:dr_lls@hotmail.com)

# Supplementary Tables

**Supplementary Table 1.** Univariate analysis of patients’ characteristics.

| Feature name | HR | Log (HR) | *p*-value | lower 95%CI | upper 95%CI |
| --- | --- | --- | --- | --- | --- |
| Site | 1.007 | 0.007 | 0.939 | -0.138 | 0.151 |
| Gender | 1.031 | 0.030 | 0.703 | -0.100 | 0.160 |
| Diameter | 1.001 | 0.001 | 0.735 | -0.003 | 0.005 |
| Age | 1.000 | -0.000 | 0.839 | -0.004 | 0.003 |

HR: hazard ratio; CI: confidence interval.

**Supplementary Table 2.** Predictive performance of model based on radiomics signature.

| Model | Accuracy | AUC | 95% CI | Sensitivity | Specificity |
| --- | --- | --- | --- | --- | --- |
| KNN | 0.48 | 0.75 | 0.54 - 0.95 | 0.36 | 1.00 |
| Random Forest | 0.87 | 0.87 | 0.68 – 1.00 | 0.88 | 0.83 |
| ExtraTrees | 0.42 | 0.63 | 0.41 – 0.84 | 0.28 | 1.00 |
| XGBoost | 0.90 | 0.83 | 0.60 – 1.00 | 0.92 | 0.83 |
| MLP | 0.81 | 0.79 | 0.58 - 0.99 | 0.84 | 0.67 |

CI: confidence interval; AUC: area under ROC curve; ROC: receiver operating characteristic; KNN: K-Nearest Neighbor; XGBoost: eXtreme Gradient Boosting; MLP: Multilayer Perceptron.
