# Supplementary material for: Machine learning-based radiomics for predicting BRAF-V600E mutations in ameloblastoma
Source: Front Immunol. 2023 Aug 14;14:1180908. doi: 10.3389/fimmu.2023.1180908 (PMC10461083; doi:10.3389/fimmu.2023.1180908)

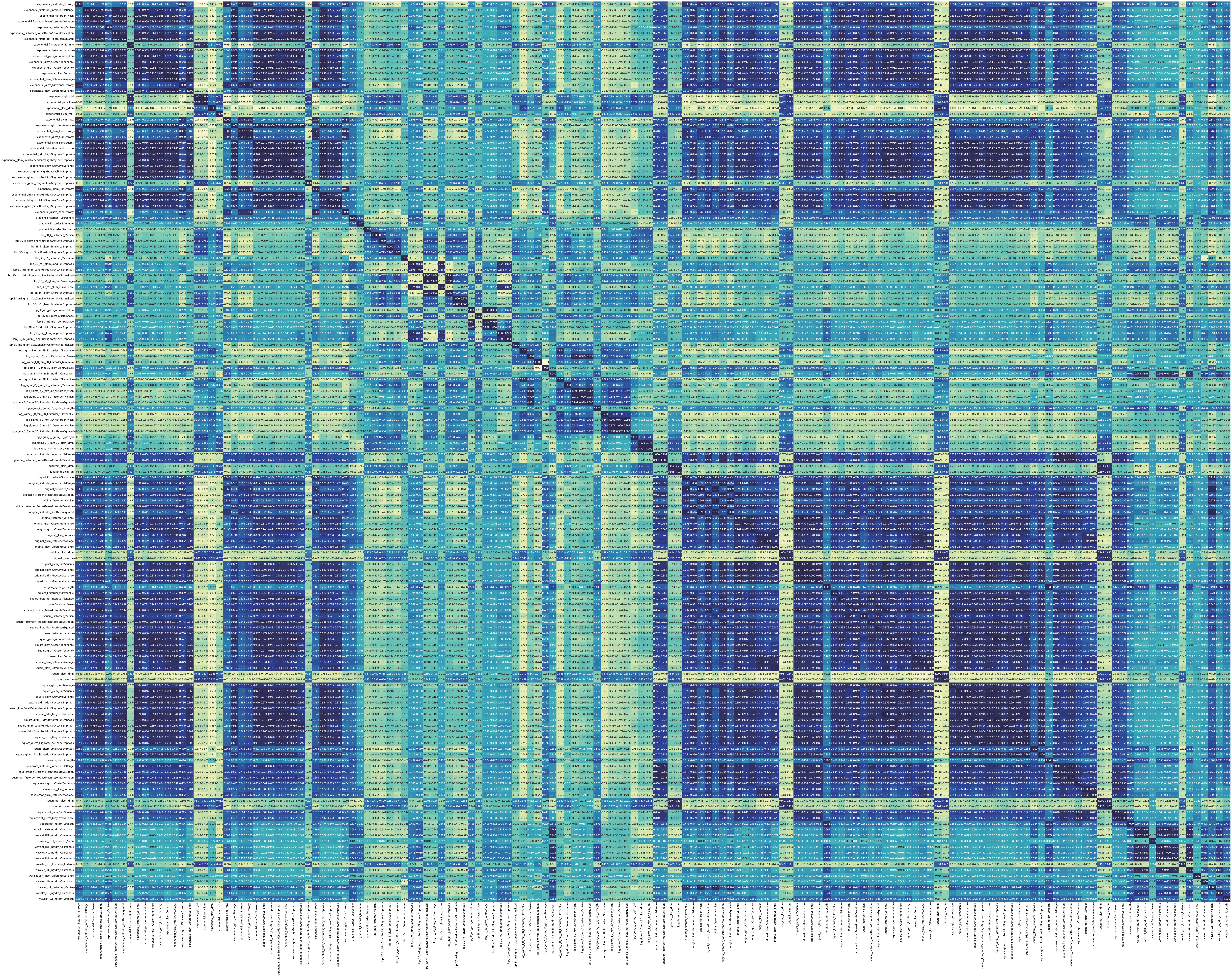

Supplement: Supplementary file 2 [file DataSheet_2.pdf]
